# Interstitial lung abnormality evaluated by an automated quantification system: prevalence and progression rate

**DOI:** 10.1186/s12931-024-02715-3

**Published:** 2024-02-06

**Authors:** Ju Hyun Oh, Grace Hyun J. Kim, Jin Woo Song

**Affiliations:** 1 Department of Pulmonology and Critical Care Medicine, Sanggye Paik Hospital, Inje University College of Medicine, Seoul, Republic of Korea; 2 Department of Radiological Sciences, David Geffen School of Medicine at UCLA, Los Angeles, USA; 3 Department of Pulmonology and Critical Care Medicine, Asan Medical Centre, University of Ulsan College of Medicine, 88, Olympic-Ro 43-gil, Songpa-gu, Seoul, 05505 Republic of Korea

**Keywords:** Interstitial lung disease, Fibrosis, Prevalence, Prognosis, Mortality

## Abstract

**Background:**

Despite the importance of recognizing interstitial lung abnormalities, screening methods using computer-based quantitative analysis are not well developed, and studies on the subject with an Asian population are rare. We aimed to identify the prevalence and progression rate of interstitial lung abnormality evaluated by an automated quantification system in the Korean population.

**Methods:**

A total of 2,890 healthy participants in a health screening program (mean age: 49 years, men: 79.5%) with serial chest computed tomography images obtained at least 5 years apart were included. Quantitative lung fibrosis scores were measured on the chest images by an automated quantification system. Interstitial lung abnormalities were defined as a score ≥ 3, and progression as any score increased above baseline.

**Results:**

Interstitial lung abnormalities were identified in 251 participants (8.6%), who were older and had a higher body mass index. The prevalence increased with age. Quantification of the follow-up images (median interval: 6.5 years) showed that 23.5% (59/251) of participants initially diagnosed with interstitial lung abnormality exhibited progression, and 11% had developed abnormalities (290/2639). Older age, higher body mass index, and higher erythrocyte sedimentation rate were independent risk factors for progression or development. The interstitial lung abnormality group had worse survival on follow-up (5-year mortality: 3.4% vs. 1.5%; *P* = 0.010).

**Conclusions:**

Interstitial lung abnormality could be identified in one-tenth of the participants, and a quarter of them showed progression. Older age, higher body mass index and higher erythrocyte sedimentation rate increased the risk of development or progression of interstitial lung abnormality.

**Supplementary Information:**

The online version contains supplementary material available at 10.1186/s12931-024-02715-3.

## Background

Interstitial lung abnormalities (ILAs) are an incidental finding of lung parenchymal abnormalities suggestive of early interstitial lung disease (ILD), which affects in more than 5% of lungs imaged by chest computed tomography (CT) [1]. In previous studies, the ILA prevalence ranged from 4 to 17% in various cohorts [2–4]. ILAs have also been associated with reduced lung function and exercise capacity, low quality of life, and increased risk of ILD occurrence and all-cause mortality [5–9]. Therefore, early detection of ILAs is increasingly being considered important [10–12].

In most studies on ILAs, radiologists or pulmonologists conducted visual assessments. However, image analysis by visual assessment is limited by inter-observer variation, reproducibility, and time consumption [2–6, 13, 14]. To overcome these limitations, computer-based image analysis methods, such as the measurement of high-attenuation areas (HAAs), density histogram evaluation, and texture-based analysis, have been performed for the radiological evaluation of ILD and ILAs [15–20]. In the Multi-Ethnic Study of Atherosclerosis (MESA), Choi et al. showed that a more extended HAA was associated with higher odds of ILA occurrence [18]. In a study including family members of patients with familial interstitial pneumonia, deep learning–based textural evaluation analysis showed a sensitivity of 84% and a specificity of 86% for detecting early ILD [15]. However, the viability of an automated quantification system (AQS) for ILA diagnosis remains unverified because factors such as slice thickness, the reconstruction algorithm used for CT images, the type of CT scanner employed, and the inspiration level of the patients potentially interfere with the results [21, 22]. In addition, most studies on ILAs have recruited non-Hispanic white or African-American participants [15–18], whereas Asian cohorts are under-represented. Therefore, in this study, we aimed to evaluate the usefulness of an automated quantitative system (AQS) in the assessment of ILAs and to use this method to determine the prevalence, progression rate, and risk factors for ILA progression in the Korean general population.

## Methods

### Study population

Overall, we screened 3,578 participants in a health screening program conducted between February 1997 and November 2007 at Asan Medical Centre (Seoul, Republic of Korea) for whom serial chest CT images were available. Participants whose chest CT images were of inadequate quality for AQS analysis (*n* = 650) owing to volume artefacts, unusable thin series, or incomplete lung coverage or those for whom serial chest CT images obtained more than 5 years apart were unavailable (*n* = 38) were excluded from the analysis. Finally, 2,890 participants were included, and none of them had a previous diagnosis of ILD. This study was approved by the Institutional Review Board of Asan Medical Center (2013 − 0957), and the requirement for informed consent was waived owing to the study’s retrospective nature.

### Clinical data

The clinical and survival data of all the participants were retrospectively collected from medical or National Health Insurance of Korea records. The results of laboratory tests, including complete blood counts, glucose, glycated haemoglobin, total protein, albumin, blood urea nitrogen, and creatinine levels, lipid profiles, and erythrocyte sedimentation rates (ESR), were also collected. Clinical evaluation and laboratory testing were performed along with the CT scan on the same day. Spirometry tests were conducted in accordance with the American Thoracic Society/European Respiratory Society guidelines [23].

### Automated quantification of CT images

All participants were scanned with a 16- or 64-detector CT scanner during breath-hold at full inspiration, with the patient in the supine position. All axial chest CT images were reconstructed at a section thickness of 1.00 or 1.25 mm, at an interval of 5 or 10 mm, using a high-spatial-frequency reconstruction algorithm (see Additional file 1). The anonymized CT images were imported and consistently labeled using the DICOM information and high-throughput tool with the prioritization of thin slices (≤ 2 mm) and relatively smooth kernel to maintain robust quantitative measurements for ILD [24, 25]. CT scans were performed at least twice sequentially (median number of CT scans: 3.0, interquartile range [IQR]: 2.0–4.0), and the first and last CT images were analysed (median interval: 78.0 months, IQR: 67.0–93.0 months). The lung parenchymal abnormalities were analysed using a computer-aided quantitative scoring system described in a previous report [26]. Briefly, automated quantitative analyses of CT images were performed in five steps: (1) de-noising of the image to reduce variation of texture features using homogenous landmarks within the CT (see the detail for Additional file 1); (2) sampling of each pixel from a 4 × 4 grid; (3) conversion of the characteristics of grid intensities into texture features; (4) classification of the texture features of pixels as specific patterns using a built-in model; and (5) calculation of the percentages corresponding to the classified pixels. Using this method, quantitative lung fibrosis (QLF) (sum of reticulation and traction bronchiectasis) ground glass opacity (GGO), honeycombing (HC) and quantitative ILD (QILD) score (sum of QLF, GGO and HC) were obtained from the CT images scores (see Additional file 2: Figure S1). ILAs were defined as a QLF score ≥ 3, and ILA progression was defined as any increase in the QLF score obtained from the follow-up CT images relative to that obtained from the initial CT images. We chose QLF ≥ 3% with consideration of technical reproducibility based on statistical evaluation, where the 3% is the sum of at least 1% of the evidence in the extent of disease and the highest outlying point of 2% [27–29].(see the detail for Additional file 1).

### Visual assessment of CT images

To evaluate the performance of the AQS for predicting visually assessed ILA, we conducted a pilot study with 307 participants for whom paired chest CT images more than 10 years apart were available. Two radiologists (H.H. and T.A.) and one pulmonologist (G.M.H), who were blinded to the clinical information associated with the CT images, visually assessed the images using the sequential reading method [30] (see the detail for Additional file 1). All the images were scored using a 3-point scale: 0 = no evidence of ILA, 1 = equivocal ILA, and 2 = ILA. ILAs were defined as non-dependent changes affecting more than 5% of any lung zone, including a reticular abnormality, ground-glass opacity (GGO), traction bronchiectasis, honeycombing, or non-emphysematous cyst [1]. Unilateral or focal GGO, unilateral or focal reticulation, or patchy GGO (< 5% of the lung) were classified as equivocal ILAs [1, 20].

### Statistical analyses

Data are presented as means ± standard deviations or numbers (%). Student’s t-test was used for continuous variables, and the chi-squared test was used to compare categorical variables. When performing the survival analysis, the follow-up period was calculated from the date of the follow-up CT scan to that of death or censoring (October 21, 2021). The Kaplan–Meier survival analysis and log-rank test were used for survival analysis. The logistic regression analysis was used to determine the risk factors for ILAs. The risk factors for ILA progression or all-cause mortality were analysed using the Cox proportional hazards analysis. The receiver-operating characteristic (ROC) curve analysis was used to evaluate the performance of the AQS system in predicting ILA by visual assessment. Inter-reader agreements were assessed with use of the weighted kappa coefficient (κ) [31]. Variables with *P*-value < 0.1 in the unadjusted analysis were included in the multivariable analysis with backward stepwise elimination. A *P*-value < 0.05 was used to indicate statistical significance. The statistical analyses were performed using the SPSS software (version 21.0; IBM Corporation, Armonk, NY, USA) or MedCalc statistical software (version 12.7.5; MedCalc Software bvba, Ostend, Belgium).

## Results

### Performance of AQS

The pilot study was conducted using the follow-up CT images of 307 participants. The mean age was 59.3 years, and 86.6% were men. Among them, 23 (7.5%) presented ILAs; 196 (63.8%), equivocal ILAs; and 88 (28.7%), no ILAs (Additional file 1). The group diagnosed with ILAs showed older age; lower total cholesterol, low-density lipoprotein (LDL), and albumin levels; and lower forced vital capacity in 1 s (FEV_1_)/forced vital capacity (FVC) than those of the no ILA groups (see Additional file 1: Table S1).

In the ROC curve analysis comparing the performance of the AQS scores in predicting ILA determined by visual assessment, the QLF score had the best predictive performance (area under the curve: 0.758, 95% confidence interval: 0.706–0.805; *P* < 0.001), compared to the other AQS scores (see Additional file 2: Figure S2). The mean QLF scores for the ILA, equivocal ILA, and no ILA groups were 3.5, 2.6, and 1.1, respectively. With the cut-off value of QLF score ≥ 3, the sensitivity, specificity, and accuracy of AQS for predicting ILA were 47.8%, 81.9%, and 79.0%, respectively (see Additional file 1: Table S2). The AQS categorized 5.7% (5/88) of the cases included in the no ILA group, 23.7% (46/196) of those in the equivocal ILA group, and 47.8% (11/23) of those in the ILA group as presenting ILA.

### Prevalence of ILA

**Fig. 1:**
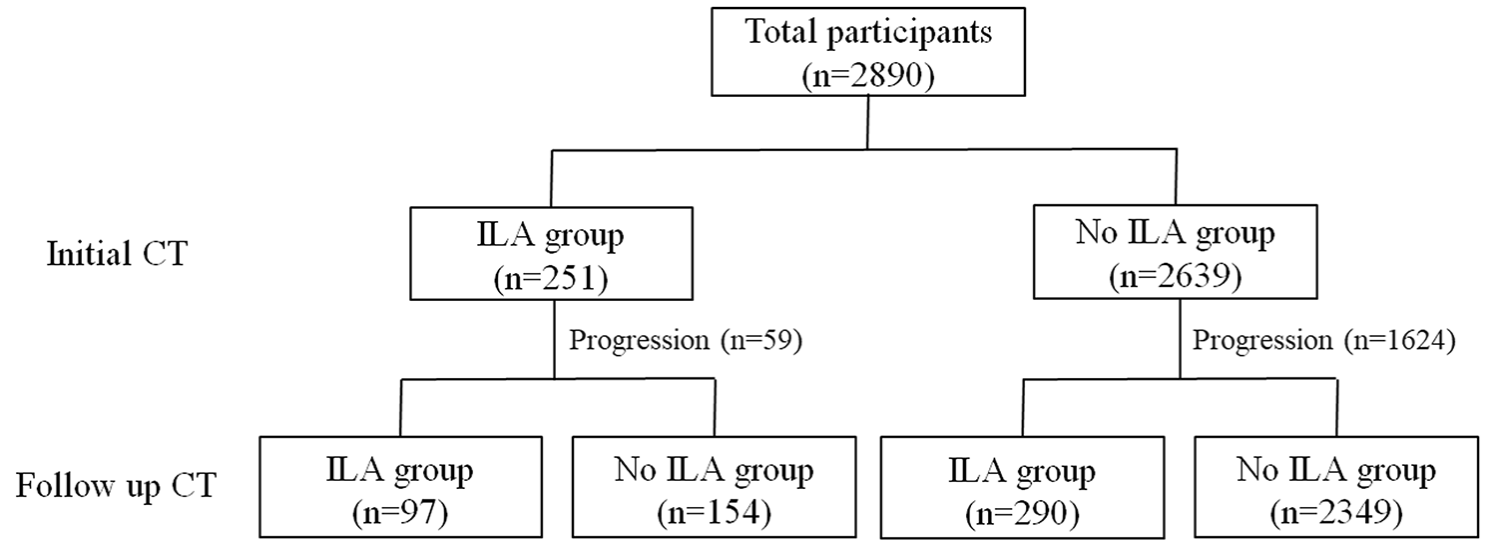
Flow chart of the participants for the initial and follow-up chest computed tomography analyses. From a total of 2,890 participants, interstitial lung abnormalities (ILAs) were identified in 251 participants (8.6%) on the baseline computed tomography (CT) images and in 387 participants (13.4%) on the follow-up CT images. On the follow-up CT images, 23.5% (59/251) of the participants included in the ILA group showed ILA progression and 10.9% (290/2639) of those in the non-ILA group showed ILA development. CT, computed tomography; ILA, interstitial lung abnormality

**Fig. 2:**
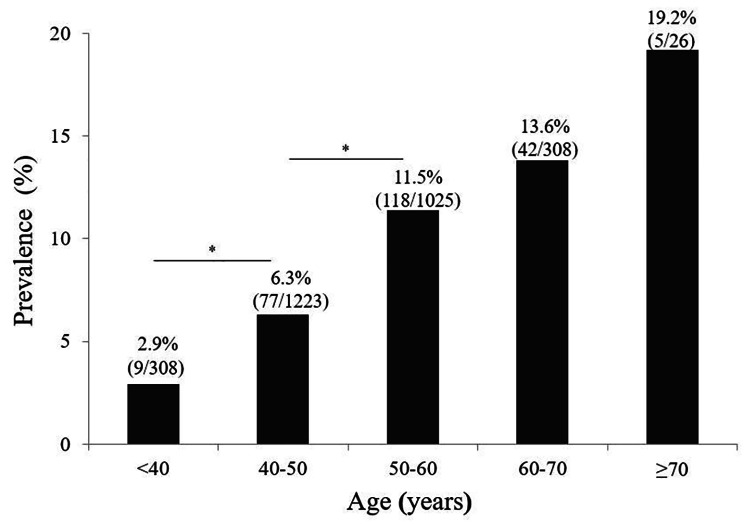
Interstitial lung abnormality prevalence stratified by age. The prevalence of interstitial lung abnormality (ILA) increased with age. ILA diagnosis was based on the baseline chest computed tomography images of the participants. *P-value < 0.05

The mean age of all the participants in the study (*n* = 2,890) was 49.4 years; 79.5% were men (see Additional file 1: Table S3). Using the AQS, ILAs were found in 251 participants (8.6%) on the baseline CT scan and in 387 participants (13.4%) on the follow-up CT scan (Fig. 1). When the participants were stratified according to age, the prevalence of ILAs increased with age: 2.9% in the participants aged under 40 years and 19.2% in those aged over 70 years (Fig. 2).

The ILA group showed older age; a higher proportion of women and non-smokers; higher body mass index (BMI) and ESR and LDL levels; and lower albumin levels than those of the no ILA group (see Additional file 1: Table S3). When evaluated on the basis of follow-up CT images, the ILA group also showed older age; a higher proportion of women and non-smokers; higher BMI and white blood cell (WBC), ESR, triglyceride, and total protein levels; and lower FVC and high-density lipoprotein (HDL) levels than those of the no ILA group (Table 1). In the multivariable logistic regression analysis, older age; female sex; higher BMI, FEV_1_/FVC, and levels of WBC and total protein; and lower FVC and HDL levels were independently associated with the presence of ILAs on the follow-up CT images (Table 2).

**Table 1:** Comparison of baseline characteristics between the interstitial lung abnormality (ILA) and no ILA groups^*^

Characteristics	Total	ILA	No ILA	*P*-value
Number of patients	2,890	387	2,503	
Age, years	55.8 ± 9.2	58.1 ± 9.1	55.5 ± 9.2	< 0.001
Male	2,297 (79.5)	292 (75.5)	2,005 (80.1)	0.042
Ever-smoker (*n* = 2,873)	2,032 (70.7)	250 (64.9)	1,782 (71.6)	0.007
BMI, kg/m^2^	24.3 ± 3.3	25.3 ± 2.9	24.1 ± 3.4	0.001
WBCs, ×10^3^/µL	5.8 ± 1.7	6.0 ± 1.8	5.8 ± 1.7	0.024
Hgb, g/dL	14.8 ± 1.4	14.8 ± 1.5	14.8 ± 1.3	0.888
Platelet, ×10^3^/µL	231.5 ± 51.1	231.0 ± 50.8	231.5 ± 51.1	0.857
ESR, mm/h	12.3 ± 10.3	14.8 ± 12.4	11.8 ± 9.9	< 0.001
HbA1c^†^, %	5.8 ± 0.8	5.8 ± 0.8	5.8 ± 0.8	0.076^†^
Total cholesterol, mg/dL	188.9 ± 34.4	188.6 ± 35.7	189.0 ± 34.1	0.818
Triglyceride, mg/dL	128.5 ± 75.9	137.1 ± 75.4	127.2 ± 75.9	0.017
HDL, mg/dL	53.6 ± 13.9	50.4 ± 13.2	54.1 ± 13.8	< 0.001
LDL, mg/dL	116.1 ± 30.3	116.9 ± 31.3	115.9 ± 30.1	0.058
Protein, g/dL	7.1 ± 0.4	7.2 ± 0.4	7.1 ± 0.4	< 0.001
Albumin, g/dL	4.2 ± 0.3	4.2 ± 0.2	4.2 ± 0.3	0.140
BUN, mg/dL	13.4 ± 3.4	13.4 ± 3.7	13.4 ± 3.4	0.726
Creatinine, mg/dL	0.9 ± 0.2	0.9 ± 0.2	0.9 ± 0.2	0.479
FVC, predicted %	89.0 ± 11.1	85.8 ± 11.1	89.5 ± 10.9	< 0.001
FEV_1_, predicted %	88.6 ± 16.1	87.6 ± 12.5	88.8 ± 12.2	0.099
FEV_1_/FVC, %	77.1 ± 3.2	77.7 ± 7.2	76.9 ± 7.3	0.075

Data are presented as means ± standard deviations or numbers (%).;ILA, interstitial lung abnormality; BMI, body mass index; WBCs, white blood cells; Hgb, haemoglobin; ESR, erythrocyte sedimentation rate; HbA1c, glycated haemoglobin; HDL, high-density lipoprotein; LDL, low-density lipoprotein; BUN, blood urea nitrogen; FVC, forced vital capacity; FEV_1_, forced vital capacity in 1 s; *evaluated on the basis of follow-up chest computed tomography (CT) images; ^†^ ILA vs. no ILA: 5.843 ± 0.765 vs. 5.769 ± 0.764, the values were rounded to one decimal place

**Table 2:** Logistic regression analysis for risk factors of interstitial lung abnormality presence^*^

Characteristics	Unadjusted analysis		Multivariable analysis	
	OR (95% CI)	*P*-value	OR (95% CI)	*P*-value
Age, years	1.035 (1.022–1.048)	< 0.001	1.042 (1.026–1.059)	< 0.001
Male	0.763 (0.594–0.982)	0.035	0.481 (0.347–0.665)	< 0.001
Ever-smoker	0.734 (0.585–0.920)	0.007	-	-
BMI, kg/m^2^	1.145 (1.102–1.189)	< 0.001	1.145 (1.092–1.200)	< 0.001
WBCs, ×10^3^/µL	1.072 (1.009–1.138)	0.024	1.071 (0.997–1.151)	0.060
Hgb, g/dL	1.006 (0.930–1.088)	0.888	-	-
Platelet, ×10^3^/µL	1.000 (0.998–1.002)	0.857	-	-
ESR, mm/h	1.024 (1.014–1.033)	< 0.001	-	-
Glucose, mg/dL	1.004 (1.000–1.008)	0.071	-	-
HbA1c, %	1.122 (0.988–1.274)	0.077	-	-
Total cholesterol, mg/dL	1.000 (0.997–1.003)	0.818	-	-
Triglyceride, mg/dL	1.002 (1.000–1.003)	0.017	-	-
HDL, mg/dL	0.979 (0.971–0.988)	< 0.001	0.984 (0.974–0.994)	0.002
LDL, mg/dL	1.001 (0.997–1.005)	0.580		
Protein, g/dL	1.754 (1.338–2.300)	< 0.001	1.667 (1.223–2.270)	0.001
Albumin, g/dL	0.730 (0.481–1.109)	0.140	-	-
BUN, mg/dL	1.006 (0.975–1.037)	0.726	-	-
Creatinine, mg/dL	0.803 (0.437–1.474)	0.479	-	-
FVC, predicted %	0.970 (0.960–0.980)	< 0.001	0.985 (0.973–0.997)	0.013
FEV_1_, predicted %	0.992 (0.983–1.001)	0.099	-	-
FEV_1_/FVC, %	1.015 (0.999–1.031)	0.075	1.019 (1.001–1.039)	0.043

OR, odds ratio; CI, confidence interval; BMI, body mass index; WBCs, white blood cells; Hgb, haemoglobin; ESR, erythrocyte sedimentation rate; HbA1c, glycated haemoglobin; HDL, high-density lipoprotein; LDL, low-density lipoprotein; BUN, blood urea nitrogen; FVC, forced vital capacity; FEV_1_, forced vital capacity in 1 s; *evaluated on the basis of follow-up chest computed tomography (CT) images

### Risk factors for ILA development or progression

Among the 251 participants initially included in the ILA group, 59 (23.5%) showed progression on the follow-up CT images (Fig. 1). Additionally, 290 (10.9%) of the 2,639 participants who did not present ILAs on the baseline CT scan showed ILA development on the follow-up CT images (Fig. 1). In the unadjusted Cox regression analysis, older age, female sex, never-smokers, higher BMI and ESR, and triglyceride and total protein levels; and lower albumin levels were associated with the development or progression of ILA (Table 3). According to the multivariable Cox regression analysis, older age; never smokers; higher BMI, ESR, and triglyceride and protein levels; and lower albumin levels were independently associated with the development or progression of ILA (Table 3).

**Table 3:** Cox regression analysis for risk factors of interstitial lung abnormality development or progression

Characteristics	Unadjusted analysis		Multivariable analysis	
	HR (95% CI)	*P*-value	HR (95% CI)	*P*-value
Age, years	1.035 (1.022–1.047)	< 0.001	1.024 (1.011–1.037)	< 0.001
Male, No. (%)	0.706 (0.549–0.907)	0.007	-	-
Ever-smoker	0.806 (0.709–0.917)	0.001	0.739 (0.580–0.941)	0.014
BMI, kg/m^2^	1.071 (1.035–1.109)	< 0.001	1.061 (1.019–1.105)	0.004
WBCs, ×10^3^/µL	1.028 (0.967–1.091)	0.378	-	-
Hgb, g/dL	0.997 (0.923–1.077)	0.942	-	-
Platelet, ×10^3^/µL	1.000 (0.998–1.002)	0.918	-	-
ESR, mm/h	1.034 (1.025–1.044)	< 0.001	1.019 (1.007–1.030)	0.002
Glucose, mg/dL	1.004 (0.999–1.009)	0.132	-	-
HbA1c, %	1.086 (0.947–1.246)	0.239	-	-
Total cholesterol, mg/dL	0.999 (0.996–1.002)	0.667	-	-
Triglyceride, mg/dL	1.002 (1.000–1.003)	0.007	1.002 (1.001–1.003)	0.002
HDL, mg/dL	0.992 (0.984–1.001)	0.087	-	-
LDL, mg/dL	1.000 (0.996–1.003)	0.901	-	-
Protein, g/dL	1.486 (1.144–1.930)	0.003	1.670 (1.140–2.446)	0.008
Albumin, g/dL	0.530 (0.364–0.770)	0.001	0.455 (0.271–0.764)	0.003
BUN, mg/dL	1.015 (0.981–1.049)	0.394	-	-
Creatinine, mg/dL	0.800 (0.425–1.504)	0.488	-	-

HR, hazard ratio; CI, confidence interval; BMI, body mass index; WBCs, white blood cells; Hgb, haemoglobin; ESR, erythrocyte sedimentation rate; HbA1c, glycated haemoglobin; HDL, high-density lipoprotein; LDL, low-density lipoprotein; BUN, blood urea nitrogen

### Association of ILA with mortality

**Fig. 3:**
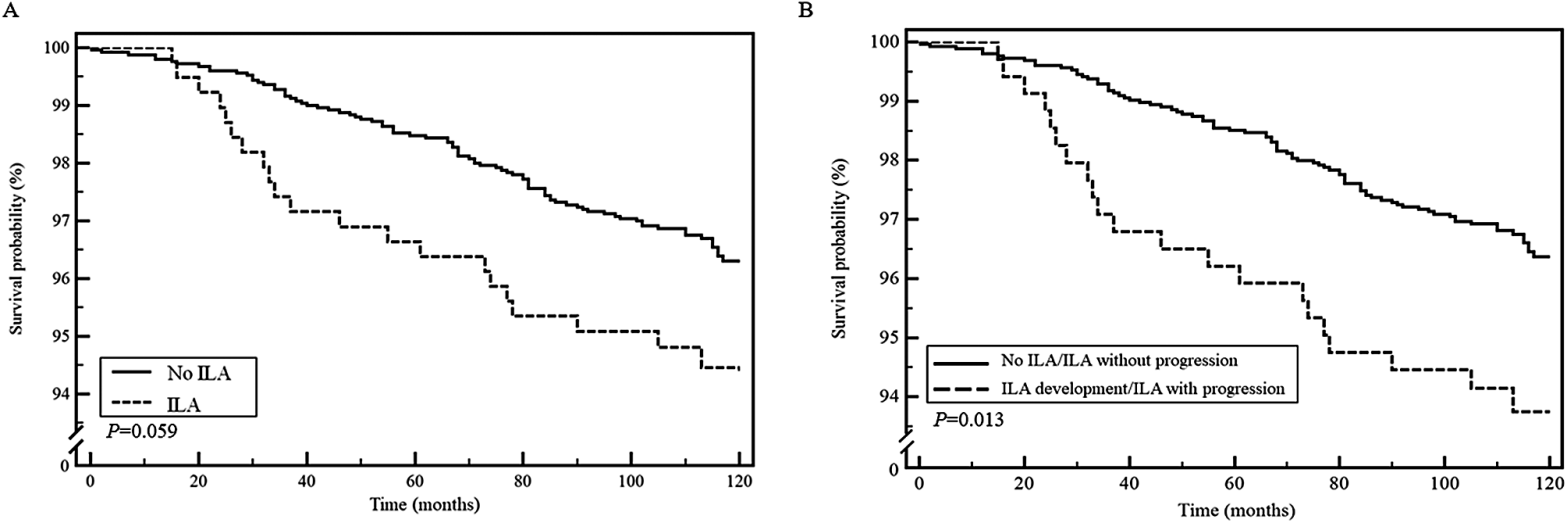
Comparison of survival outcomes. **A**. Comparison of Kaplan–Meier survival curves between the interstitial lung abnormality (ILA) and no ILA groups. **B**. Comparison of Kaplan–Meier survival curves between participants with and without ILA development or progression. The ILA group showed higher mortality than that of the no ILA group (5-year mortality: 1.5% vs. 3.4%; 10-year mortality: 3.4% vs. 5.4%; log-rank *P* = 0.059). Those who exhibited ILA progression or development also showed higher mortality than that of those without ILA or progression (5-year mortality: 1.5% vs. 3.8%; 10-year mortality: 3.4% vs. 6.1%; log-rank *P* = 0.013). ILA, interstitial lung abnormality

During the 10-year follow-up period from the date of the last CT scan (median: 116.0 months, IQR: 109.0–120.0 months), 3.7% (107/2890) of the participants died. The ILA group had worse survival than did the non-ILA group (5-year mortality: 3.4% vs. 1.5%; 10-year mortality: 5.4% vs. 3.4%; mean survival time: 116.2 ± 0.8 vs. 118.0 ± 0.2 months, log-rank *P* = 0.059; Fig. 3A). Those who presented ILA progression or development showed significantly worse survival than that of those with no ILA or ILA without progression (5-year mortality: 3.8% vs. 1.5%; 10-year mortality 6.1% vs. 3.4%; mean survival time: 115.7 ± 0.9 vs. 118.1 ± 0.2 months, log-rank, *P* = 0.013) (Fig. 3B). In the ILA group, older age, ever-smokers, higher ESR, and lower albumin levels were independent risk factors for mortality according to the results of the multivariable Cox analysis (Table 4).

**Table 4:** Cox regression analysis for risk factors of mortality in participants with interstitial lung abnormality^*^

Characteristics	Unadjusted analysis		Multivariable analysis	
	HR (95% CI)	*P*-value	HR (95% CI)	*P*-value
Age, years	1.085 (1.037–1.137)	< 0.001	1.062 (1.012–1.115)	0.015
Male	1.976 (0.582–6.707)	0.275	-	-
Ever-smokers	3.306 (0.974–11.222)	0.055	3.802 (1.044–13.842)	0.043
BMI, kg/m^2^	1.003 (0.864–1.165)	0.967	-	-
WBCs, ×10^3^/µL	1.226 (1.042–1.443)	0.014	-	-
Hgb, g/dL	0.838 (0.637–1.103)	0.208	-	-
Platelet, ×10^3^/µL	0.999 (0.990–1.007)	0.739	-	-
ESR, mm/h	1.044 (1.022–1.066)	< 0.001	1.036 (1.005–1.067)	0.021
Glucose, mg/dL	1.008 (0.990–1.026)	0.401	-	-
HbA1c, %	1.476 (0.997–2.187)	0.052	-	-
Total cholesterol, mg/dL	0.987 (0.974–0.999)	0.041	-	-
Triglyceride, mg/dL	1.000 (0.995–1.006)	0.916	-	-
HDL, mg/dL	0.942 (0.902–0.984)	0.007	-	-
LDL, mg/dL	0.990 (0.977–1.004)	0.175	-	-
Protein, g/dL	0.498 (0.165–1.506)	0.217	-	-
Albumin, g/dL	0.061 (0.020–0.193)	< 0.001	0.173 (0.040–0.738)	0.018
BUN, mg/dL	1.070 (0.965–1.186)	0.202	-	-
Creatinine, mg/dL	2.822 (0.553–14.401)	0.212	-	-
FVC, predicted %	0.960 (0.919–1.003)	0.069	-	-
FEV_1_, predicted %	0.981 (0.945–1.019)	0.324	-	-
FEV_1_/FVC, %	0.805 (0.709–0.912)	0.001	-	-

HR, hazard ratio; CI, confidence interval; BMI, body mass index; WBCs, white blood cells; Hgb, haemoglobin; ESR, erythrocyte sedimentation rate; HbA1c, glycated haemoglobin; HDL, high-density lipoprotein; LDL, low-density lipoprotein; BUN, blood urea nitrogen; FVC, forced vital capacity; FEV_1_, forced vital capacity in 1 s; *evaluated on the basis of follow-up chest computed tomography (CT) images

## Discussion

Our study suggests that the AQS has a good performance for detecting ILAs and may be useful in identifying their presence and progression. The ILA prevalence determined by the AQS in the Korean population was 8.6% on baseline CT images and increased with age. ILA progression was identified in approximately one-fourth of the participants, and ILA development in approximately 10% of the participants during the roughly 6-year follow-up. Furthermore, participants with ILAs had worse survival than did those without ILAs.

In previous studies, ILA prevalence ranged from 3 to 10% in the general population [2, 6, 32, 33] and from 7 to 17% in smokers and in cohorts screened for lung cancer [4, 5, 20]. In our study, the ILA prevalence was 8.6%, which is similar to that reported in previous population-based studies. However, in some studies including Asian cohorts, prevalence of ILA have been reported to be around 3%, which is lower than individuals in Western countries [34–36]. Results on the prevalence of ILA may be influenced by a number of factors, including cohort characteristics, reader experience, and assessment methods. The study by Tsushima et al. including a Japanese general population with a prevalence of ILA of approximately 3%, had a mean age of 57.2 years, indicating a younger cohort compared with other ILA studies, and the definition of ILA in this study included honeycombing, interlobular septal thickening, ground glass opacity, ill-defined subpleural line, and combined pulmonary fibrosis and emphysema, which differs from recent ILA studies [36]. Chae et al. applied deep learning-based texture analysis to assess ILA in a lung cancer screening cohort, and reported a relatively low prevalence of ILA of 4% [34]. However, this lower prevalence may be due to the prior exclusion of images containing lung destruction, underinspiration, or dependent and passive atelectasis, which may have influenced the assessment of ILA by AQS. Thus, the variation in the prevalence of ILA does not indicate an inadequate application of quantitative assessments, but rather highlight the complexity of the ILA assessment, which require the detection of subtle lung parenchymal abnormalities. Therefore, we suggest that given that ILA is an indicator of early ILD, an automated quantification method may have useful clinical value in rapidly and objectively detecting lung parenchymal abnormalities that may be associated with early ILD, rather than focusing solely on the accurate discrimination of ILA diagnosed by visual assessment. This is supported by our findings that the ILA group identified by AQS has a worse prognosis than those without. The utility of using automated quantification techniques to identify early ILDs has also been demonstrated in the example of high attenuation area [17].

The ILA progression rate varies from 30 to 70%, depending on the cohort and observation periods [2–4, 6, 20, 37]. However, our results showed a lower progression rate than those of other studies including the general population with similar follow-up periods [2, 37]. In the study by Araki et al. that included the Framingham Heart Study cohort, ILA progression was reported in 43% (23/53) of participants with ILAs during the 6-year follow-up period [2]. A recent longitudinal ILA study of a large Chinese population also reported a progression rate of 43.6% over 4 years [37]. Considering that older age is an important risk factor for ILA progression [6], the between-cohort age difference may have caused this discrepancy, as the participants in the present study were younger than those in other studies; the mean age of the ILA group was 52.8 years (at baseline CT) in our study and 60 or 70 years in previous studies [2, 6, 20]. Additionally, our study assessed ILA progression according to changes in the QLF scores, whereas other studies of various lung parenchymal abnormalities, including GGO, reticulation, non-emphysematous cysts, and honeycombing, evaluated ILA progression qualitatively (side-by-side comparison) using visual assessment [2, 6, 20, 37].

Older age, polymorphisms in the *MUC5B* promoter, and air pollution are reportedly risk factors for ILA [5, 17, 32, 38]. Our study also revealed that the ILA group had lower HDL levels than did the non-ILA group, and lower HDL levels were associated with ILAs in the multivariable analysis. Previous studies have suggested a role for plasma lipid profiles and lipoproteins in ILD [39, 40]. In a study that included 6,814 MESA participants, Podolanczuk et al. reported that lower plasma HDL levels were associated with a higher HAA (percentage change in HAA per standard deviation of HDL: -2.12; 95% CI: -2.79 to -1.44; *P* < 0.001, adjusted by demographics, smoking, and inflammatory biomarkers) [39]. In another study that included 266 patients with IPF, Barochia et al. showed that serum levels of small HDL particles were negatively correlated with the gender–age–physiology (GAP) index (*r* = − 0.29, *P* = 0.03) and that higher serum levels of small HDL particles were associated with a lower risk of death or lung transplantation (odds ratio [OR]: 0.9; 95% CI: 0.82–0.97 at 1 year, adjusted by race, BMI, GAP index, and treatment status) in patients with IPF [40]. These findings suggest that HDL or its components might have protective effects in lung parenchymal injury and fibrosis.

Furthermore, in our study, higher triglyceride levels and BMI were independently associated with ILA development or progression. The results of previous reports support our findings [6, 39]. Podolanczuk et al. showed that each standard deviation increment in triglyceride levels was associated with a 2.81% increment in metalloproteinase-7 levels (95% CI: 0.30–5.37; *P* = 0.03) [39], which had been associated with ILD progression in previous studies [41, 42]. In a study including 3,167 participants from the Age, Gene/Environment Susceptibility (AGES)–Reykjavik cohort, Putman et al. reported that BMI was significantly associated with ILA progression (OR: 1.06; 95% CI: 1.02–1.09; *P* = 0.001) in a multivariable logistic regression analysis [6]. In addition, in a study involving 6,784 participants enrolled in MESA, Anderson et al. showed that every doubling in pericardial adipose tissue volume was associated with increased odds of ILAs by 20% (OR: 1.2; 95% CI: 0.98–1.5; *P* = 0.07) [43]. On the basis of these results, adiposity might be associated with parenchymal lung injury [6, 43], and adipose-derived pro-inflammatory cytokines such as interleukin-6 and monocyte chemoattractant protein–1 have been implicated in ILD pathogenesis [44–47].

In this study, we revealed that ILAs were associated with a higher risk of all-cause mortality, and that participants with progressive ILA (ILA progression or development) had worse survival than did those with no ILA or ILA without progression. These results are comparable with those from previous studies [2, 6, 33]. A study including the general population as well as smoker cohorts showed that the absolute mortality rate varied between the cohorts owing to differences in cohort characteristics and follow-up periods [33]. However, compared with the no ILA group, the ILA group was consistently associated with an increased risk of mortality in all the cohorts (hazard ratio [HR]: 1.3–2.7, adjusted by age, sex, race, BMI, and smoking status) [33]. Furthermore, in the AGES–Reykjavik cohort, ILA progression was associated with an increased risk of death (HR: 1.9; 95% CI: 1.3–2.8; *P* = 0.0009; adjusted by age, sex, BMI, and smoking history) [6]. Therefore, considering these results, even if there are no significant clinical manifestations for accidentally discovered ILA cases, these patients are expected to have a poor prognosis, and progression should be closely monitored and addressed with appropriate treatment interventions.

This study has some limitations. First, since the QLF score, which is the sum of traction bronchiectasis and reticulation, was only considered for ILA evaluation by AQS, the effects of other parenchymal lung abnormalities (including GGO, honeycombing, and non-emphysematous cysts) could not be evaluated. However, considering that reticulation and traction bronchiectasis have been associated with ILA prognosis in previous studies [6, 37], QLF scores may have more important clinical implications for ILA evaluation. Second, the prevalence of ILA may have been overestimated. Visual assessment of ILA excludes dependent, focal, or unilateral lung parenchymal changes among the lung parenchymal abnormalities [1, 2, 33]. In our study, the location and distribution of parenchymal changes were not considered in the AQS-based assessment. Therefore, some cases classified as equivocal ILA by visual assessment may have been misclassified as ILA by the AQS. However, our study suggests that ILA assessment by the AQS could provide results that are comparable to those obtained by visual assessment. Third, in this study, we only presented all-cause mortality because information on specific causes of death was not available. As shown in the AGES-Reykjavik study, we might expect the increased risk of respiratory death in ILA [33]. However, data on cause of death were also not available in other studies because of difficulties in collecting this information [33]. Finally, our study was a retrospective study conducted in a single centre and only included participants from a health screening program with serial chest CT images. Therefore, there is a possibility of selection bias. This issue seems to have contributed to some different findings in our study compared with other reports; female sex and ever-smoking status have not been identified as independent risk factors in previous ILA studies [3, 6, 32, 48]. However, in our study, they showed a significant association with the presence or progression of ILA, respectively. At the initial CT or during follow-up, most participants diagnosed with chronic lung diseases such as ILD/ILA or those with severe symptoms may have been referred to a respiratory clinic and did not undergo follow-up CT scans as part of the health screening examination. Therefore, it is possible that males or smokers at high risk of chronic lung disease were excluded from the cohort. Despite these limitations, our study is valuable in that it included a relatively large number of participants with a wide age range (under 40 to over 70 years) who were followed up for an extended period.

In conclusion, our study suggests that an AQS might be a useful tool for detecting ILAs, and that the prevalence, progression rate, and prognostic impact in the Korean population are comparable to what has been reported in previous studies.

### Electronic supplementary material

Below is the link to the electronic supplementary material.

Supplementary Material 1

Supplementary Material 2

## Data Availability

The datasets from this study are available from the corresponding author upon request.

## Abbreviations

AQS: Automated quantification system
BMI: Body mass index
CT: Computed tomography
ESR: Erythrocyte sedimentation rates
FEV1: Forced vital capacity in 1 s
FVC: Forced vital capacity
GGO: Ground-glass opacity
HAAs: High-attenuation areas
HbA1c: Glycated haemoglobin
HDL: High-density lipoprotein
ILA: Interstitial lung abnormalities
ILD: Interstitial lung disease
LDL: Low-density lipoprotein
QLF: Quantitative lung fibrosis
ROC: Receiver-operating characteristic
WBCs: White blood cells
