# Supplementary material for: Interstitial lung abnormality evaluated by an automated quantification system: prevalence and progression rate
Source: Respir Res. 2024 Feb 6;25:78. doi: 10.1186/s12931-024-02715-3 (PMC10848490; doi:10.1186/s12931-024-02715-3)
Supplement: Supplementary file 1 — Supplementary Material 1 [file 12931_2024_2715_MOESM1_ESM.docx]

**CT acquisition protocol**

Each participant was scanned with one of the following multi-detector CT scanners: Somatom Sensation 16, Definition AS, Definition AS+, Definition Flash, or Definition Edge (Siemens Healthineers, Erlangen, Germany); LightSpeed 16, LightSpeed VCT, or Discovery CT750 HD (GE Healthcare, Milwaukee, Wis). CT scans were conducted in full inspiration in the supine position with one of the following protocols: standard radiation dose with contrast media or standard radiation or low radiation dose without contrast media. Standard-dose non-contrast CT was gained using 120 kVp and 100 reference mAs or 100–300 mA, and low-dose non-contrast CT using 120 kVp and 20 reference mAs or 50 mA. Standard-dose contrast-enhanced CT was performed using 120 kVp and 100 reference mAs or 100–300 mA, following intravenous administration of 90–120 mL of 300–370 mgI/mL non-ionic contrast at a rate of 2.5 mL/s using a power injector with a scan delay of 50 seconds. All axial images were reconstructed at a section thickness of 1.00 or 1.25 mm at intervals of 5 or 10mm, using a high-spatial-frequency reconstruction algorithm

**Preliminary Studies for new applications**

1. Simulated Low Dose data & CT Image Reconstruction

We simulated low dose sinogram data by factoring the ratio of reduction into the original sinogram data according to Zabic’s algorithm [1]. The algorithm accounts for both the quantum noise due to photon statistics and the electronic detector noise components of the data acquisition process. For the detail, a low dose signal is obtained: an approximated low dose. For reconstruction of HRCT images, we used the standard helical weighted-filtered back projection methods [2, 3]

1. Denoise-Adaptive denoise in Machine Learning: Currently, we use the adaptive denoise based on

imaging characteristics in total variation. Denoise is based on Aujol and Gill’s iterative total variance algorithm and the dual norm in the Besov topological space with a nonlinear project, which has the flexibility of various noise and low dose scans like lung screening population [4, 5] After adaptive denoise, by tuning the parameter (delta, δ), the robust QLF scores were obtained across different doses level.

Gilles’ and extension Aujol’s Algorithm [4, 5]

1. Initialization:

u_0_ = v_0_ = 0

1. Iterations:

w _n+1_ = P_δ BG_ ( f − u _n_ − v _n_ ) ; noised image

v _n+1_ = P_µ BG_ ( f − u _n_ − w _n+1_ ) ; texture image

u _n+1_ = f − v _n+1_ − w _n+1_ − P_μ BG_ ( f − v _n+1_ − w _n+1_ ) ; geometric image

1. Stopping test: stop if

max( | u _n+1_ − u _n_ | , | v _n+1_ − v _n_ | , | w_n+1_  − w _n_ |) ≤ ε

where *u, v* and *w* represent the geometric, texture, and noised images, respectively. And the sum of *u* and *v* image is denoised image. *PBG* is a non-linear projection using a discrete version of gradient operator and divergence operator in adjacent voxels and solved by a fixed-point method. And δ represents the amount of noise and λ represents the accuracy of algorithm. The sum of *u, v*, and *w* is approximately equal to original CT image if the algorithm converges. For the reasonable noise CT image, the threshold of delta is generally set to 50 and increase as the noisy metric of uniform areas increases [6]. For the dose simulation study, which obtained from the raw data, we were able to preserve the equivalent level of QLF scores in the dose reduction of 100%, 30%, and 15% level from the diagnostic doses. QLF score preserves in different dose levels (30% and 15% relative to conventional doses, respectively) by adaptive denoise technique, where the denoise parameter (delta) was estimated by noise from the uniform areas of trachea and background air of each CT scan [6] (Supplementary Figure 1). Twelve subjects’ HRCT scans were reconstructed systematically reconstructed by original dose 100mAs, and 50mAs. The case ID is ranged from 201 to 212 (Supplementary Figure 2). Adaptive denoise of parameters worked reasonably well for the original dose, 100mAs, and 50mAs for the delta of 50, 60, and 70, respectively. One case (#202) had to require special attention due to the high BMI of the absolute dose reduction (Supplement Figure 2). After using the personalized noise level in adaptive denoise, the differences were within 1%. From this pilot study, we implemented the adaptive denoise per patient-specific CT image using the homogeneous landmarks of the air in the outside of chest (background air), aorta, and trachea [6].


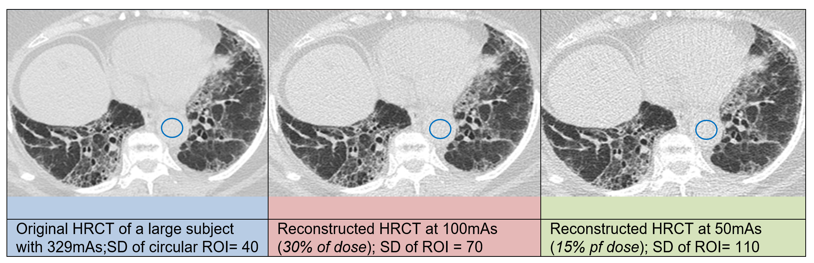
Supplement Figure 1. Stabilized QLF score in Whole lung

Supplement Figure 2. Bar graphs of QLF scores and difference by patient and dose level

**QLF threshold for defining ILA**

We chose QLF≥3% with consideration of technical reproducibility based on statistical evaluation, where the 3% is the sum of at least 1% of the evidence in the extent of disease and the highest outlying point of 2%. In our previous study, we ran the reproducibility and visualized the difference using the Bland-Altman plot. The largest absolute difference was close to 2% for the non-volumetric scan: (1.96*$\sqrt{2})*$0.72=2.77*0.72= 1.99) [7, 8].

**Visual evaluation of the interstitial lung abnormality (ILA)**

Two radiologists (Hiroto Hatabu, Tetsuro Araki [Department of Radiology, Brigham and Women's Hospital, Harvard Medical School, USA], with 40 years and 19 years of experience, respectively) and one pulmonologist (Gary M Hunninghake [Division of Pulmonary and Critical Care, Brigham and Women's Hospital, Harvard Medical School, USA] with 20 years of experience), who were blinded to the clinical information associated with the CT images, visually assessed the images using the sequential reading method. In the sequential reading method, reader 1 would review the all images and assign a score of 0, 1 or 2 (0=no evidence of ILA, 1=equivocal ILA, and 2=ILA). CT scans including those scored 1 or 2 and a random selection of approximately 20% of the normal scans (score 0), would be provided to reader 2, who was blinded to the initial interpretation. Finally, reader 3, who was blinded to the interpretations of readers 1 and 2, made the final decision by providing an opinion on scans that disagreed between readers 1 and 2.

Out of 307 cases, 86 cases were classified as normal (score 0), 194 cases were classified as equivocal ILA (score 1), and 27 cases were classified as suspected ILA (score 2) by reader 1. And the re-evaluation by reader 2 was performed for 238 cases of initial 307 cases (221 cases with score of 1 or 2, and 17 cases with a score of 0). Between reader 1 and reader 2, there was disagreement in 54.2% (n=129) of cases, requiring a third review. Finally, reader 3 provided an opinion for the final decision.

Of 27 cases with a score of 2 by reader 1, 20 cases had a final score of 2 and the remaining 7 cases had a final score of 1. Of 194 cases with a score of 1 by reader 1, 189 cases had a final score of 1 by reader 3, with the remaining 3 cases having a final score of 2 and 2 cases having a final score of 0. However, no case initially given a score of 0 was changed. Kappa agreements for ILA status (ILA, Equivocal ILA, No ILA) between the three readers and the final results are detailed in the following table.

| **Readers** | **Cohen weighted κ value (95% CI)** | |
| --- | --- | --- |
| Reader 1 vs Final | | 0.930 (0.891-0.968) |
| Reader 2 vs Final | | 0.239 (0.106-0.317) |
| Reader 3 vs Final | | 0.597 (0.430-0.765) |

1. Žabić S, Wang Q, Morton T, Brown KM: **A low dose simulation tool for CT systems with energy integrating detectors.** *Medical physics* 2013, **40:**031102.

2. Young S, Kim HJG, Ko MM, Ko WW, Flores C, McNitt‐Gray MF: **Variability in CT lung‐nodule volumetry: Effects of dose reduction and reconstruction methods.** *Medical physics* 2015, **42:**2679-2689.

3. Chong DY, Kim HJ, Lo P, Young S, McNitt-Gray MF, Abtin F, Goldin JG, Brown MS: **Robustness-Driven Feature Selection in Classification of Fibrotic Interstitial Lung Disease Patterns in Computed Tomography Using 3D Texture Features.** *IEEE Trans Med Imaging* 2016, **35:**144-157.

4. Gilles J: **Noisy image decomposition: a new structure, texture and noise model based on local adaptivity.** *Journal of Mathematical Imaging and Vision* 2007, **28:**285-295.

5. Aujol J-F, Gilboa G, Chan T, Osher S: **Structure-texture image decomposition—modeling, algorithms, and parameter selection.** *International journal of computer vision* 2006, **67:**111-136.

6. Kim HJ, Li G, Gjertson D, Elashoff R, Shah SK, Ochs R, Vasunilashorn F, Abtin F, Brown MS, Goldin JG: **Classification of parenchymal abnormality in scleroderma lung using a novel approach to denoise images collected via a multicenter study.** *Academic radiology* 2008, **15:**1004-1016.

7. Kim HJ, Brown MS, Elashoff R, Li G, Gjertson DW, Lynch DA, Strollo DC, Kleerup E, Chong D, Shah SK, et al: **Quantitative texture-based assessment of one-year changes in fibrotic reticular patterns on HRCT in scleroderma lung disease treated with oral cyclophosphamide.** *European Radiology* 2011, **21:**2455-2465.

8. Obuchowski NA, Reeves AP, Huang EP, Wang XF, Buckler AJ, Kim HJ, Barnhart HX, Jackson EF, Giger ML, Pennello G, et al: **Quantitative imaging biomarkers: a review of statistical methods for computer algorithm comparisons.** *Stat Methods Med Res* 2015, **24:**68-106.

9. Kundel HL, Polansky M: **Measurement of observer agreement.** *Radiology* 2003, **228:**303-308.

**Table S1.** Comparison of baseline characteristics of participants according to the presence of interstitial lung abnormalities evaluated by visual assessment^*^

| **Characteristics** | **Total** | **ILA** | **Equivocal ILA** | **No ILA** | ***P*-value**^†^ |
| --- | --- | --- | --- | --- | --- |
| Number of patients | 307 | 23 | 196 | 88 |  |
| Age, years | 59.3 ± 8.1 | 67.1 ± 6.7 | 59.6 ± 8.4 | 56.5 ± 7.0 | <0.001 |
| Male | 266 (86.6) | 20 (86.9) | 174 (88.8) | 72 (81.8) | 0.759 |
| Ever-smokers | 241 (78.5) | 16 (69.6) | 155 (79.1) | 70 (79.5) | 0.400 |
| BMI, kg/m^2^ | 24.5 ± 2.9 | 23.8 ± 2.9 | 24.5 ± 2.8 | 24.8 ± 3.1 | 0.163 |
| WBCs, ×10^3^/µL | 5.6 ± 1.6 | 5.9 ± 1.6 | 5.7 ± 1.5 | 5.6 ± 1.9 | 0.322 |
| Hgb, g/dL | 14.8 ± 1.3 | 14.3 ± 1.2 | 14.9 ± 1.3 | 14.7 ± 1.4 | 0.303 |
| Platelet, ×10^3^/µL | 222.1 ± 44.6 | 215.8 ± 38.1 | 224.5 ± 46.0 | 221.2 ± 44.9 | 0.598 |
| ESR, mm/h | 11.1 ± 8.8 | 15.9 ± 14.0 | 10.7 ± 8.1 | 10.8 ± 7.6 | 0.094 |
| HbA1c, % | 5.8 ± 0.7 | 5.8 ± 0.6 | 5.8 ± 0.7 | 5.7 ± 0.6 | 0.466 |
| Total cholesterol, mg/dL | 184.9 ± 34.7 | 173.7 ± 32.6 | 184.1 ± 34.3 | 190.7 ± 34.1 | 0.027 |
| Triglyceride, mg/dL | 119.0 ± 55.0 | 108.6 ± 63.2 | 124.4 ± 60.6 | 118.4 ± 50.9 | 0.726 |
| HDL, mg/dL | 52.5 ± 13.8 | 50.5 ± 12.5 | 52.3 ± 13.1 | 53.4 ± 15.2 | 0.472 |
| LDL, mg/dL | 114.3 ± 30.5 | 106.7 ± 25.9 | 112.8 ± 30.3 | 118.9 ± 30.2 | 0.048 |
| Protein, g/dL | 7.1 ± 0.4 | 7.1 ± 0.4 | 7.0 ± 0.5 | 7.1 ± 0.4 | 0.731 |
| Albumin, g/dL | 4.2 ± 0.3 | 4.0 ± 0.3 | 4.2 ± 0.3 | 4.2 ± 0.3 | 0.040 |
| BUN, mg/dL | 13.6 ± 3.5 | 13.5 ± 3.6 | 13.5 ± 3.4 | 13.5 ± 3.6 | 0.943 |
| Creatinine, mg/dL | 0.9 ± 0.2 | 0.9 ± 0.2 | 0.9 ± 0.2 | 0.9 ± 0.2 | 0.952 |
| FVC, predicted % | 96.9 ± 13.2 | 96.8 ± 17.2 | 96.1 ± 13.1 | 98.9 ± 11.2 | 0.450 |
| FEV_1_, predicted % | 102.3 ± 14.8 | 99.7 ± 19.2 | 101.2 ± 14.8 | 104.6 ± 13.0 | 0.254 |
| FEV_1_/FVC | 75.7 ± 6.7 | 71.8 ± 8.4 | 75.2 ± 6.8 | 77.3 ± 4.9 | 0.001 |

Data are presented as means ± standard deviations or numbers (%); ILA, interstitial lung abnormalities; BMI, body mass index; WBCs, white blood cells; Hgb, haemoglobin; HbA1c, glycated haemoglobin; ESR, erythrocyte sedimentation rate; HDL, high-density lipoprotein; LDL, low-density lipoprotein; BUN, blood urea nitrogen; FVC, forced vital capacity; FEV_1_, forced vital capacity in 1 s; * evaluated on the basis of follow-up chest computed tomography (CT) images; ^†^ *P*-value between ILA and no ILA

**Table S2.** Predictive performance of automated quantification system scores for interstitial lung abnormalities determined by visual assessment

| **Cut-off value** | **Sensitivity** | **Specificity** | **Accuracy** |
| --- | --- | --- | --- |
| QLF score≥2 | 73.9% | 65.0% | 65.7% |
| QLF score≥3 | 47.8% | 81.9% | 79.0% |
| QLF score≥4 | 26.1% | 89.4% | 84.6% |

QLF, quantitative lung fibrosis

**Table S3.** Comparison of baseline characteristics between interstitial lung abnormality (ILA) and no ILA groups^*^

| **Characteristics** | **Total** | **ILA** | **No ILA** | ***P*-value** |
| --- | --- | --- | --- | --- |
| Number of patients | 2,890 | 251 | 2,639 |  |
| Age, years | 49.4 ± 8.3 | 52.8 ± 7.8 | 49.1 ± 8.2 | <0.001 |
| Male, % | 2299 (79.5) | 292 (75.5) | 2007 (80.1) | 0.034 |
| Ever-smoker (n=2,537) | 1631 (72) | 126 (57.8) | 1643 (70.8) | <0.001 |
| BMI, kg/m^2^ | 24.4 ± 3.2 | 25.6 ± 3.2 | 24.3 ± 3.2 | <0.001 |
| WBCs, ×10^3^/µL | 5.9 ± 1.7 | 6.1±1.7 | 5.9±1.7 | 0.214 |
| Hgb, g/dL | 15.1 ± 1.4 | 15.1±1.4 | 15.1±1.4 | 0.647 |
| Platelet, ×10^3^/µL | 238.9 ± 51.2 | 237.7±51.1 | 239.1±51.3 | 0.679 |
| ESR, mm/h | 11.4 ± 10.2 | 14.4±11.5 | 11.1±10.0 | <0.001 |
| HbA1c, % | 15.1 ± 1.4 | 5.6±0.7 | 5.6±0.7 | 0.055 |
| Total cholesterol, mg/dL | 197.0 ± 33.3 | 201.1±36.8 | 196.6±32.9 | 0.064 |
| Triglyceride, mg/dL | 134.6 ± 77.7 | 136.5±75.3 | 134.4±77.9 | 0.675 |
| HDL, mg/dL | 53.1 ± 13.4 | 51.6±13.1 | 53.3±13.5 | 0.057 |
| LDL, mg/dL | 128.8 ± 29.9 | 132.9±31.3 | 128.4±29.8 | 0.034 |
| Protein, g/dL | 7.2 ± 0.4 | 7.2±0.4 | 7.2±0.4 | 0.208 |
| Albumin, g/dL | 4.3 ± 0.3 | 4.2±0.3 | 4.3±0.3 | <0.001 |
| BUN, mg/dL | 13.4 ± 3.2 | 13.5±3.2 | 13.3±3.2 | 0.444 |

Data are presented as means ± standard deviations or numbers (%); ILA, interstitial lung abnormality; BMI, body mass index; WBCs, white blood cells; Hgb, haemoglobin; HbA1c, glycated haemoglobin; ESR, erythrocyte sedimentation rate; HDL, high-density lipoprotein; LDL, low-density lipoprotein; BUN, blood urea nitrogen; *evaluated on the basis of baseline chest computed tomography (CT) images
