# Supplementary material for: Interstitial lung abnormality evaluated by an automated quantification system: prevalence and progression rate
Source: Respir Res. 2024 Feb 6;25:78. doi: 10.1186/s12931-024-02715-3 (PMC10848490; doi:10.1186/s12931-024-02715-3)
Supplement: Supplementary file 2 — Supplementary Material 2 [file 12931_2024_2715_MOESM2_ESM.docx]

**Figure S1.** Analysis of CT images by automated quantification system.


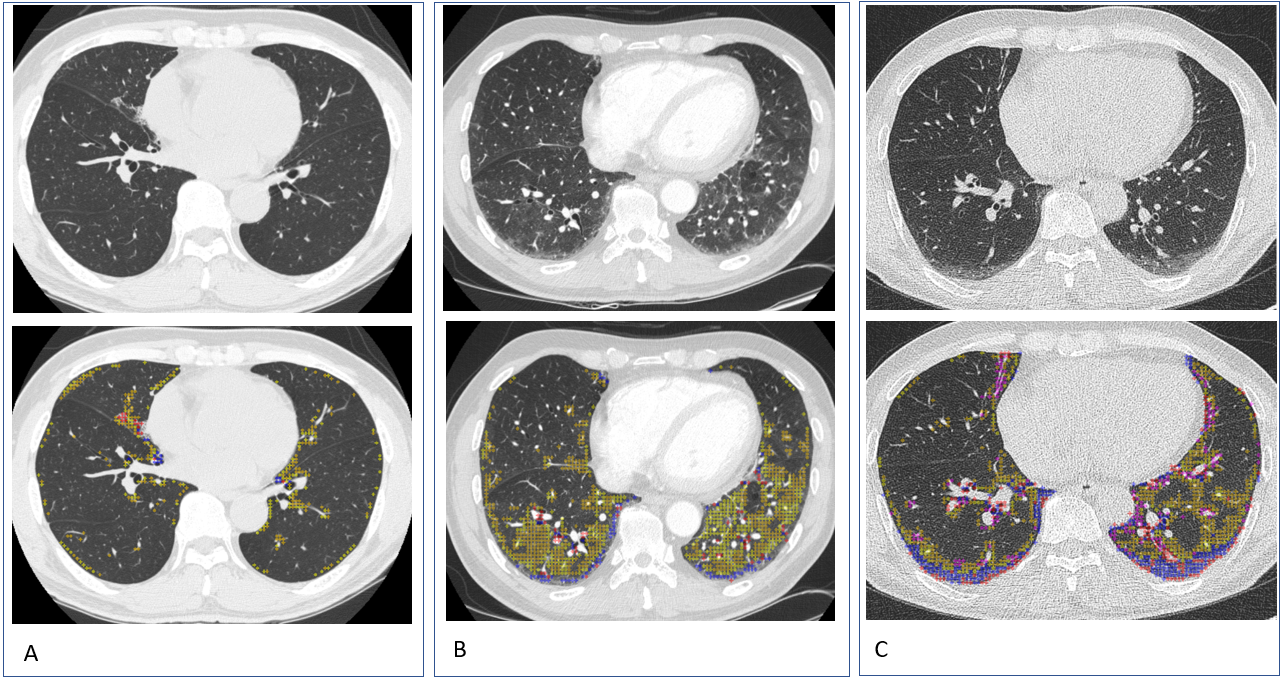


Axial chest CT images with quantitative classification where the red and blue dots are annotations for the quantitative lung fibrosis (QLF), yellow dots for quantitative ground glass (QGG), and pink dots for honeycombing (QHC). A) a 65-year-old male at baseline CT scan with QLF=0.3%, QILD=7.2% (QGG=6.9%, HC=0%); B) a 66-year-old male at follow-up CT scan with QLF=4.6%, QILD = 29.6% (QGG=21.4, QHC=0%); C) a 54-year-old male at follow-up CT scan, with low doses and sharp kernel where QLF=10.8%, QILD=29.7% (QGG=15.4%, HC=3.5%), NL = 70.3%.

QLF, quantitative lung fibrosis; HC, honeycombing; GGO, ground glass opacity; QILD, quantitative interstitial lung disease; NL, normal lung

**Figure S2.** Receiver-operating characteristic curve analysis of automated quantification system scores for predicting interstitial lung abnormalities determined by visual assessment


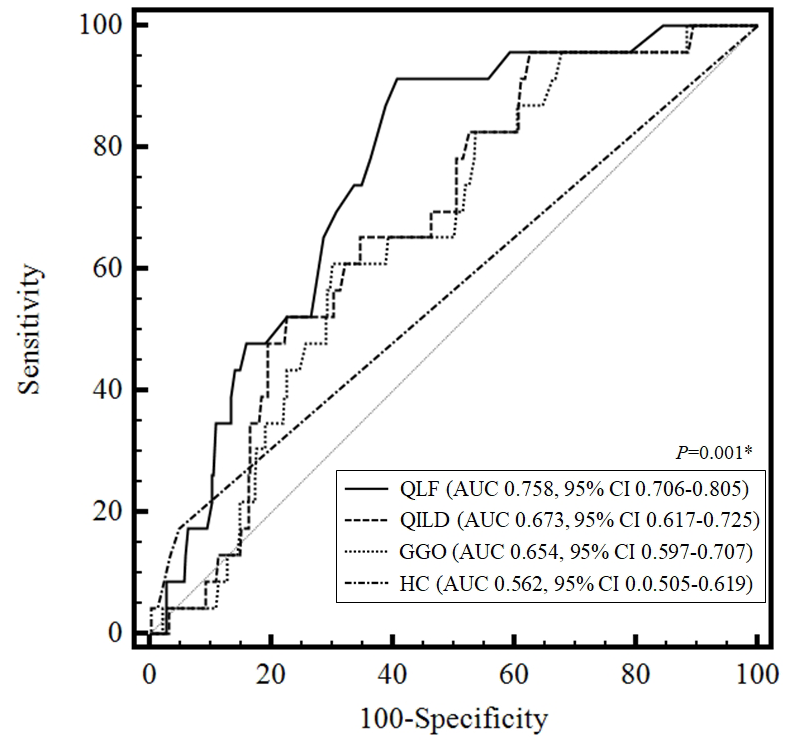


In the ROC curve analysis comparing the performance of the AQS scores in predicting ILA determined by visual assessment, the QLF score had the best predictive performance (area under the curve: 0.758, 95% confidence interval: 0.706-0.805; *P*<0.001), compared to the other AQS scores.

AUC, area under the curve; CI, confidence interval; QLF, quantitative lung fibrosis; HC, honeycombing; GGO, ground glass opacity; QILD, quantitative interstitial lung disease; * *P*-value between QLF and QILD
